# Development and validation of a kinematic hindlimb cycling model for rats

**DOI:** 10.1038/s41598-025-29285-8

**Published:** 2025-11-27

**Authors:** Hannah M. Sweatland, Eric Pettit, Heet Patel, Neeley de la Mata, Christine F. Conover, Emily J. Griffis, Joshua F. Yarrow, Matthew A. Schiefer, Warren E. Dixon

**Affiliations:** 1 Department of Mechanical and Aerospace Engineering, University of Florida, Gainesville, FL USA; 2 Department of Biomedical Engineering, University of Florida, Gainesville, FL USA; 3 Department of Neuroscience, University of Florida, Gainesville, FL USA; 4 Malcom Randall Veterans Affairs Medical Center, North Florida/South Georgia Veterans Health System, Gainesville, FL USA; 5 Eastern Colorado Geriatrics Research, Education, and Clinical Center, VA Eastern Colorado Health Care System, Aurora, CO USA; 6 Division of Geriatric Medicine, Department of Medicine, University of Colorado Anschutz Medical Campus, Aurora, CO USA; 7 West Virginia University School of Medicine, Morgantown, WV USA

**Keywords:** Kinematic analysis, Functional electrical stimulation, Physical rehabilitation, Rats, Spinal cord injury, Biomedical engineering, Mechanical engineering

## Abstract

Functional electrical stimulation (FES) bicycle training is a physical rehabilitation technique used to promote muscle recovery and/or cardiorespiratory health in persons with lower extremity impairment due to neurologic injury. FES cycling may also increase bone mineral density (BMD) in such populations, although no consensus exists that supports FES-induced skeletal improvement, in-part due to the extended 9–12 + month duration necessary to detect BMD gain in humans and the multitude of FES parameter permutations that require optimization to improve bone strength and/or reduce fracture risk, which may differ from those needed to improve muscle or cardiovascular fitness. Rodent models have been used in FES studies because musculoskeletal changes are phenotypically like humans but occur over an accelerated time course that permits more rapid identification of potentially efficacious FES parameters. To gain accelerated understanding of FES-cycling in humans, we performed a kinematic analysis of the rat hindlimb with a fixed hip location and variable foot location as the pedal of the bicycle rotated about the crank. Based on this analysis, an FES pattern was developed for the femoral and sciatic nerves to produce forward (clockwise) or reverse (counterclockwise) motion of the crank. These modeled FES patterns were validated in nine experiments that used 743 unique stimulation trials conducted in anesthetized male and female rodents. Such insights represent initial steps to facilitate closed-loop FES control of cycling in rats, which will help to refine rehabilitation strategies to promote bone and muscle recovery in rodent models and ultimately people.

## Introduction

Due in part to musculoskeletal unloading that occurs after neurologic injury, disuse or neurogenic osteoporosis can develop and increase bone fracture risk^1^. One prevalent example of this is spinal cord injury (SCI), which dramatically increases bone loss^2^ and fracture risk^3^ at the distal femur and proximal tibial areas near the knee. This bone weakness can lead to other comorbidities such as respiratory illness, pressure ulcers, and urinary tract infections that worsen mortality risk^4^. Functional electrical stimulation (FES)-induced cycling is one method of stimulating peripheral nerves and muscles in a manner that (1) does not require central nervous system (CNS) input to reload affected limbs; (2) improves muscle mass; and (3) promotes sensorimotor recovery in some individuals with SCI^5–7^. However, improvements in bone mineral density (BMD), bone microstructure, and bone strength have been difficult to demonstrate in response to FES in persons with SCI^1^. For example, some FES-cycling clinical studies have shown minor BMD gains^8–10^, while others report no trabecular or cortical bone gain^11–13^. Many factors can be varied when designing FES schemes to promote musculoskeletal recovery, such as frequency and duration of training sessions, pedaling cadence, and the frequency, amplitude, and pulse width of electrical stimulation. It is difficult to predict how the variation of these factors would impact bone structure and bone mechanical characteristics. Moreover, because of the relatively slow rate of bone metabolism in humans^14^, years-long clinical trials would be needed to optimize such factors when developing FES-cycling protocols to improve bone parameters in persons after SCI.

One approach to expedite the identification of optimal FES-cycling protocols for bone recovery is to reverse-translate the design of existing human FES-bicycle systems^15^ to develop an FES-cycling system for use in rodent models, which are an accepted model of human bone diseases^16^. For example, the severe contusion SCI model closely reproduces that pathophysiology of severe traumatic SCI in humans^17^ and findings from several labs have reported that bone phenotypic changes in rats subjected to various models of contusion SCI^18–23^ closely reproduce those occurring in humans after severe traumatic SCI^24^. However, because bone loss and regain occurs 25–50 + times faster in rats with SCI^18,19^ when compared to people with SCI^2,25^, the back-translation of an FES-bicycle may allow for higher-throughput experimentation than is currently possible with human-subject testing, using a model with musculoskeletal pathophysiology comparable to humans^17^. A passive-isokinetic motor-driven bicycle for rats was previously developed^26^ and tested^27^ and has been shown to promote near-complete trabecular and cortical bone recovery at the distal femur and proximal tibia in a rat severe SCI model within several weeks of training^28,29^. However, in these experiments, recovery of hindlimb muscle mass did not occur in response to passive-isokinetic (motorized) bicycle training after severe SCI and the use of FES was not previously considered in rodent cycling models, despite the ability of FES to promote improvement in various aspects of skeletal and cardiac muscle performance in humans with SCI^30–32^.

An important feature of human FES-cycling is the automatic switching of FES between muscle groups so that each muscle is only stimulated in the region of the crank cycle where it is kinematically efficient to do so and to avoid simultaneous co-contraction of antagonist muscle groups that would limit productive cycling (e.g., co-contraction of knee extensor and knee flexor groups), which delays the onset of fatigue and enables longer bicycle training sessions^33^. Before developing a closed-loop FES bicycle for rats, a model that informs the closed-loop controller needs to be developed. Based on a similar analysis in humans^34^, this paper details the kinematic model and stimulation patterns, which can be used to facilitate closed-loop clockwise and counterclockwise FES-cycling for rats in future experiments, accounting for the rat quadrupedal locomotion and unique hindlimb kinematics^35^. The theoretical kinematic model was validated in a series of 743 experimental trials conducted on anesthetized neurologically intact rats. Future work can use the developed FES patterns to design a closed-loop controller for continuous FES-cycling, which will facilitate experiments for bone and muscle recovery or for improvement in other health parameters.

**Fig. 1:**
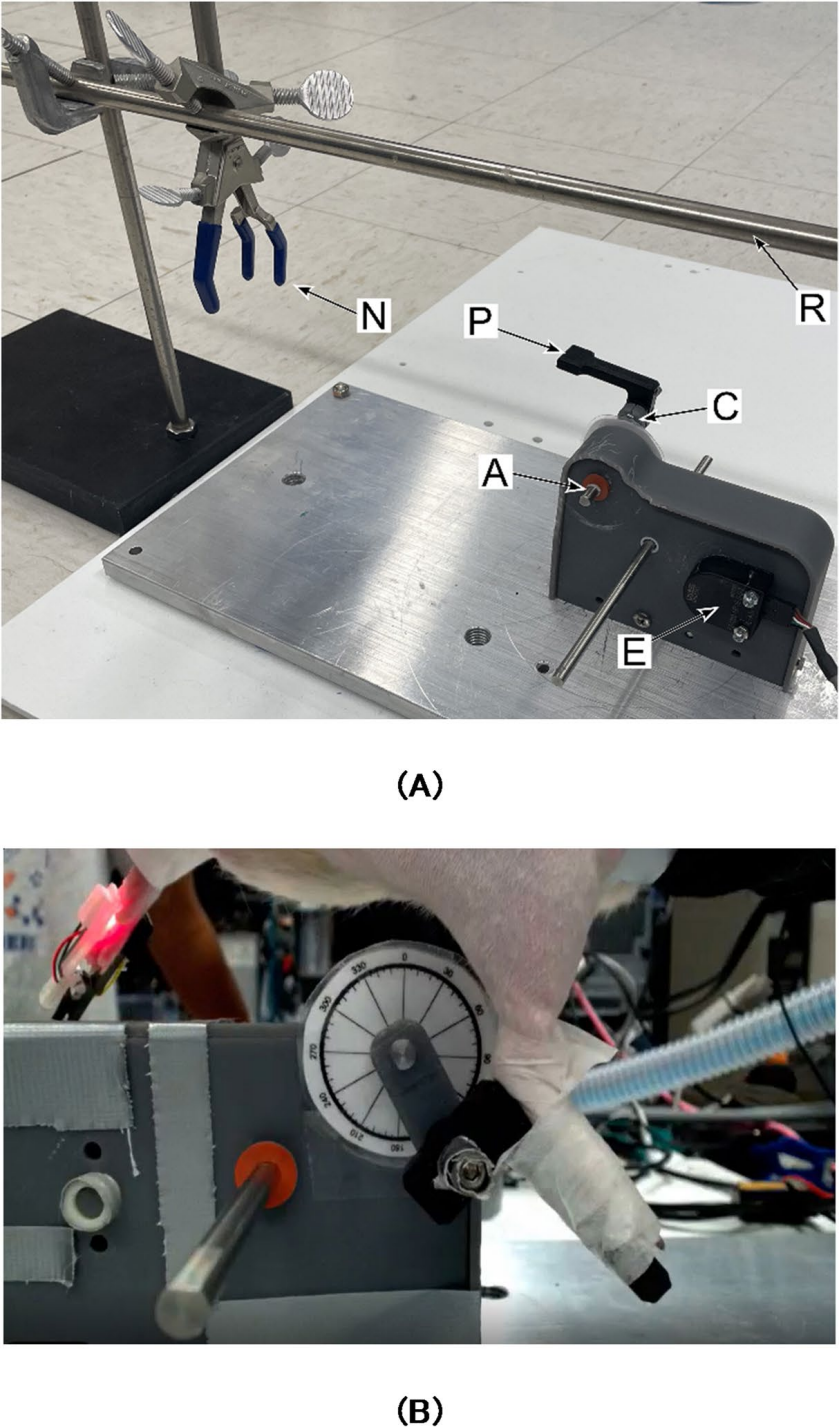
(**A**) The rodent bicycle includes a pedal (P), the crank arm (C), the crank axle (A), and the encoder (E). Not shown are gears that transmit rotation from the crank axle to the encoder. Accompanying the bicycle is a bodyweight supporting rod (R) that extends above the bicycle and a nosecone holder (N) for delivering anesthesia. (**B**) Experimental setup with rodent harnessed to support rod (R) showing the lateral view of the rodent’s right leg and foot on the pedal (P).

## Methods

### Development of the rodent bicycle

A stationary bicycle system was developed for this application and is shown in Fig. 1A. The design was based on a passive rat bicycle^26,27^ and includes pedals of sufficient length to support the hind paws of an adult male rat and a horizontal rod mounted above the animal to support the bodyweight within a sling. The bicycle was equipped with an encoder (US Digital E2) to measure the crank position in degrees, mimicking modern FES-cycling systems for human riders, with data acquired via a data acquisition board (Quanser Q8-USB) controlled by Simulink (Mathworks). Figure 1B shows the experimental setup with the rat harnessed to the mounting rod (not visible) from the right side of the bicycle.

### Development of the kinematic model

Quadrupedal locomotion has similarities to human bipedal locomotion^36^. Because the design of the rat bicycle is based on a previously developed rehabilitative bicycle for human riders, a kinematic modeling-based analysis like the one by Bellman^34^ was performed to model the angles and velocities of the hip, knee, and ankle joints of a rat during cycling. The coupled kinematic chains fixed to the ground frame had one degree-of-freedom. Therefore, with known link lengths, each joint angle was calculated based on the crank angle measured by an encoder attached to the crank.

**Fig. 2:**
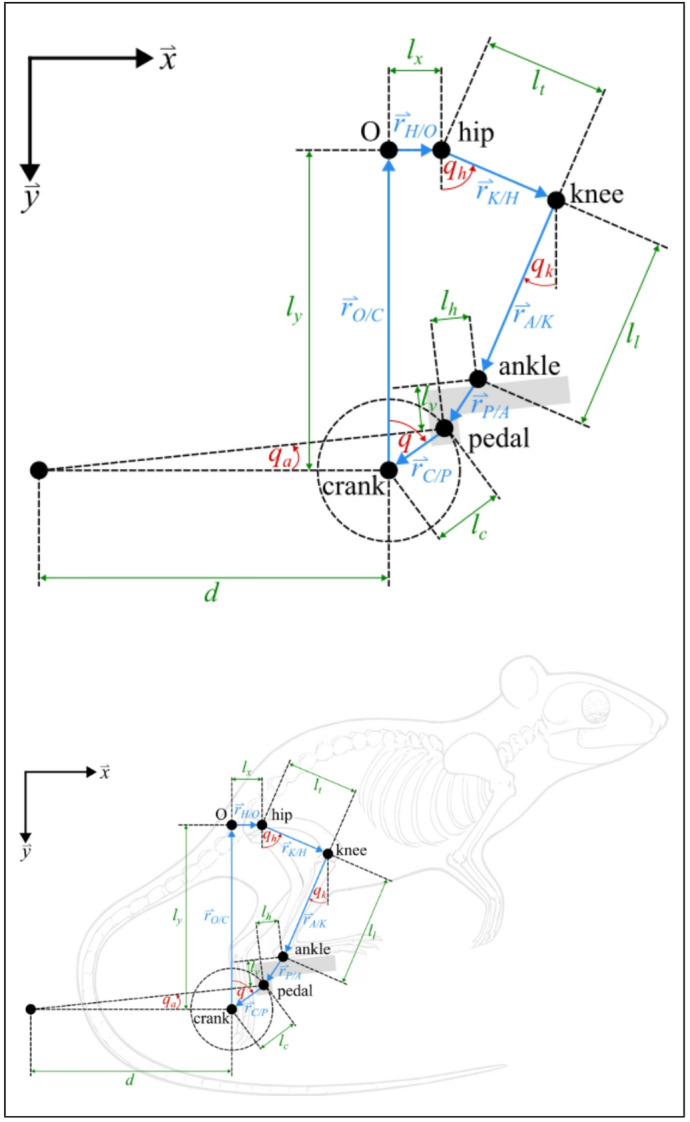
Free body diagram (FBD) of the closed kinematic chain of the right leg (top) and superimposed on a rat to show how the FBD aligns with the anatomy (bottom).

As indicated in Fig. 2, the closed-loop kinematic chain for each side of the system can be written as

1 $$\:{\overrightarrow{r}}_{H/O}+{\overrightarrow{r}}_{K/H}\left(q\left(t\right)\right)+{\overrightarrow{r}}_{A/K}\left(q\left(t\right)\right)+{\overrightarrow{r}}_{P/A}\left(q\left(t\right)\right)+{\overrightarrow{r}}_{C/P}\left(q\left(t\right)\right)+{\overrightarrow{r}}_{O/C}=\overrightarrow{0}$$

Where $\:q\in\:Q$ is the measured crank angle, $\:Q\subseteq\:\mathbb{R}$ denotes the set of possible crank angles (0-360°), $\:{\overrightarrow{r}}_{H/O}\in\:{\mathbb{R}}^{2}$ represents the vector from the fixed origin point *O* to the hip joint, $\:{\overrightarrow{r}}_{K/H},\:{\overrightarrow{r}}_{A/K},\:{\overrightarrow{r}}_{P/A},{\overrightarrow{r}}_{C/P}\in\:Q\to\:{\mathbb{R}}^{2}$ represent the vectors between the hip and knee joints, knee and ankle joints, ankle and pedal joints, and pedal and crank joints, respectively, and $\:{\overrightarrow{r}}_{O/C}\in\:{\mathbb{R}}^{2}$ represents the vector from the crank center to the origin point. The following development considers the right-hand side of the system. Knowing that the left-hand side of the bicycle crank is 180° offset from the right, the joint angles and stimulation regions for the left hindlimb can be found using the same approach.

Based on the coordinate system in Fig. 2, each term in (1) can be represented as

$$\:{\overrightarrow{r}}_{H/O}={l}_{x}\overrightarrow{x}+0\overrightarrow{y}$$

$$\:{\overrightarrow{r}}_{K/H}\left(q\left(t\right)\right)={-l}_{t}\mathrm{sin}\left({q}_{h}\left(q\left(t\right)\right)\right)\overrightarrow{x}+{l}_{t}\mathrm{cos}\left({q}_{h}\left(q\left(t\right)\right)\right)\overrightarrow{y}$$

$$\:{\overrightarrow{r}}_{A/K}\left(q\left(t\right)\right)={-l}_{l}\mathrm{sin}\left({q}_{k}\left(q\left(t\right)\right)\right)\overrightarrow{x}+{l}_{l}\mathrm{cos}\left({q}_{k}\left(q\left(t\right)\right)\right)\overrightarrow{y}$$

$$\:{\overrightarrow{r}}_{P/A}\left(q\left(t\right)\right)=\left({l}_{v}\mathrm{sin}\left({q}_{a}\left(q\left(t\right)\right)\right)-{l}_{h}\mathrm{cos}\left({q}_{a}\left(q\left(t\right)\right)\right)\right)\overrightarrow{x}-\left({l}_{h}\mathrm{sin}\left({q}_{a}\left(q\left(t\right)\right)\right)-{l}_{v}\mathrm{cos}\left({q}_{a}\left(q\left(t\right)\right)\right)\right)\overrightarrow{y}$$

$$\:{\overrightarrow{r}}_{C/P}\left(q\left(t\right)\right)={-l}_{C}\mathrm{sin}\left(\left(q\left(t\right)\right)\right)\overrightarrow{x}+{l}_{C}\mathrm{cos}\left(\left(q\left(t\right)\right)\right)\overrightarrow{y}$$

$$\:{\overrightarrow{r}}_{O/C}=0\overrightarrow{x}+{l}_{y}\overrightarrow{y}.$$

Where hip, knee, and ankle angles are denoted $\:{q}_{h},\:{q}_{k},\:{q}_{a}:\:Q\to\:\mathbb{R}$, the fixed lengths of the thigh, knee, and crank are denoted $\:{l}_{t},\:{l}_{l},\:{l}_{c}\in\:{\mathbb{R}}_{>0}$, the components of the distance between the ankle and pedal joint are denoted $\:{l}_{h},\:{l}_{v}\in\:{\mathbb{R}}_{>0}$, and the fixed $\:x$ and $\:y$ components of the distance between the crank joint and hip joint are denoted $\:{l}_{x},\:{l}_{y}\in\:{\mathbb{R}}_{>0}$. Solving for the hip, knee, and ankle angles in terms of $\:q$ provides insight into the relationship between each link and helps determine the regions of the crank cycle where FES of each muscle will cause forward (clockwise) or reverse (counterclockwise) motion of the crank.

### Joint angles

Joint angles $\:{q}_{h},\:{q}_{k},\:$and $\:{q}_{a}$ can each be found based on the measured crank angle $\:q$. Unlike in Bellman^34^, the ankle angle $\:{q}_{a}$ varies over the crank cycle and can be calculated based on the geometry of the cycle as

2 $$\:{q}_{a}=-\mathrm{a}\mathrm{r}\mathrm{c}\mathrm{t}\mathrm{a}\mathrm{n}\left(\frac{{l}_{c}\:\mathrm{c}\mathrm{o}\mathrm{s}\left(q\right)}{{l}_{c}\mathrm{sin}\left(q\right)+d}\right)$$

where $\:{l}_{c}\in\:{\mathbb{R}}_{>0}$ is the length of the crank and $\:d\in\:{\mathbb{R}}_{>0}$ is the horizontal distance between the crankshaft and the mounting point of the rod that keeps the pedal in the correct orientation.

To find the remaining joint angles, we parameterize the system in terms of crank angle $\:q$. Based on Crane and Duffy^37^, $\:{q}_{h}$ can be expressed as

3 $$\:{q}_{h}=\pm\:\mathrm{a}\mathrm{r}\mathrm{c}\mathrm{c}\mathrm{o}\mathrm{s}\left(-\frac{{k}_{3}\left(q\right)}{\sqrt{{k}_{1}{\left(q\right)}^{2}+{k}_{2}{\left(q\right)}^{2}}}\right)+\gamma\:\left(q\right)$$

where

$$\:{k}_{1}\left(q\right)\triangleq\:2{l}_{t}{l}_{h}\mathrm{c}\mathrm{o}\mathrm{s}\left({q}_{a}\right)-2{l}_{t}{l}_{v}\mathrm{s}\mathrm{i}\mathrm{n}\left({q}_{a}\right)+2{l}_{t}{l}_{c}\mathrm{s}\mathrm{i}\mathrm{n}\left(q\right)-2{l}_{t}{l}_{x}$$

$$\:{k}_{2}\left(q\right)\triangleq\:-2{l}_{t}{l}_{h}\mathrm{s}\mathrm{i}\mathrm{n}\left({q}_{a}\right)+2{l}_{t}{l}_{v}\mathrm{c}\mathrm{o}\mathrm{s}\left({q}_{a}\right)+2{l}_{t}{l}_{c}\mathrm{c}\mathrm{o}\mathrm{s}\left(q\right)-2{l}_{t}{l}_{y}.$$

$$\:{k}_{3}\left(q\right)\triangleq\:{l}_{t}^{2}+{l}_{h}^{2}+{l}_{v}^{2}+{l}_{c}^{2}+{l}_{x}^{2}+{l}_{y}^{2}-{l}_{l}^{2}$$

$$\:-2{l}_{h}{l}_{v}cos\left({q}_{a}\right)sin\left({q}_{a}\right)+2{l}_{h}{l}_{c}cos\left({q}_{a}\right)sin\left(q\right)$$

$$\:-\:2{l}_{h}{l}_{x}cos\left({q}_{a}\right)-2{l}_{v}{l}_{c}sin\left({q}_{a}\right)sin\left(q\right)$$

$$\:+\:2{l}_{v}{l}_{x}sin\left({q}_{a}\right)-2{l}_{c}{l}_{x}sin\left(q\right)$$

$$\:-\:2{l}_{h}{l}_{v}sin\left({q}_{a}\right)cos\left({q}_{a}\right)-2{l}_{h}{l}_{c}sin\left({q}_{a}\right)cos\left(q\right)$$

$$\:+\:2{l}_{h}{l}_{y}sin\left({q}_{a}\right)+\:2{l}_{v}{l}_{c}cos\left({q}_{a}\right)cos\left(q\right)$$

$$\:-\:2{l}_{v}{l}_{y}cos\left({q}_{a}\right)-2{l}_{c}{l}_{y}cos\left(q\right)$$

and $\:\gamma\:\triangleq\:\mathrm{a}\mathrm{r}\mathrm{c}\mathrm{t}\mathrm{a}\mathrm{n}\left(\frac{{k}_{1}\left(q\right)}{{k}_{2}\left(q\right)}\right)$. There are two solutions to (3), but the positive solution of $\:{q}_{h}$ corresponds to hyperextension of the hip joint and is therefore ignored. The knee angle $\:{q}_{k}$ is a function of $\:{q}_{h},\:{q}_{a},$ and $\:q$ and can be written as

$$\:{q}_{k}=arctan\left(\frac{{l}_{x}-{l}_{t}\mathrm{s}\mathrm{i}\mathrm{n}\left({q}_{h}\right)-{l}_{h}\mathrm{c}\mathrm{o}\mathrm{s}\left({q}_{a}\right)-{l}_{v}\mathrm{s}\mathrm{i}\mathrm{n}\left({q}_{a}\right)-{l}_{c}\mathrm{s}\mathrm{i}\mathrm{n}\left(q\right)}{{l}_{y}-{l}_{t}\mathrm{c}\mathrm{o}\mathrm{s}\left({q}_{h}\right)+{l}_{h}\mathrm{s}\mathrm{i}\mathrm{n}\left({q}_{a}\right)-{l}_{v}\mathrm{c}\mathrm{o}\mathrm{s}\left({q}_{a}\right)-{l}_{c}\mathrm{c}\mathrm{o}\mathrm{s}\left(q\right)}\right)$$

where $\:{l}_{x}\in\:\mathbb{R}$ and $\:{l}_{y},\:{l}_{t},{l}_{h},{l}_{v}\in\:{\mathbb{R}}_{>0}$ are the known constant lengths of each link in the kinematic chain.

#### Joint velocities

Ankle, hip, and knee joint velocities are functions of the crank position and velocity. Velocity transformation terms for the ankle, hip, and knee joints relate the velocity of the crank to the velocity of each joint and are denoted $\:{S}_{a},\:{S}_{h},\:{S}_{k}:\:Q\to\:\mathbb{R}$, respectively. The ankle velocity transformation term $\:{S}_{a}$ can be calculated from the time derivative of (2) as

$$\:{S}_{a}\triangleq\:\frac{{l}_{c}^{2}+{dl}_{c}\mathrm{s}\mathrm{i}\mathrm{n}\left(q\right)}{{\mathrm{sec}}^{2}\left({q}_{a}\right){\left({l}_{c}\mathrm{sin}\left(q\right)+d\right)}^{2}}.$$

Taking the time derivative of (1), solving for $\:{\dot{q}}_{h}$ and $\:{\dot{q}}_{k}$ in terms of $\:\dot{q}$, and substituting $\:{S}_{a}$ yields the relationships between the velocity of the crank and the velocities of the hip and knee joints

4 $$\:\left[\begin{array}{c}{\dot{q}}_{h}\left(q,\dot{q}\right)\\\:{\dot{q}}_{k}\left(q,\dot{q}\right)\end{array}\right]=\left[\begin{array}{c}{S}_{h}\left(q\right)\\\:{S}_{k}\left(q\right)\end{array}\right]\dot{q}$$

where $\:{S}_{h}$and $\:{S}_{k}$ are defined as

$$\:{S}_{h}\triangleq\:\frac{-sin\left({q}_{k}\right)\left({l}_{c}\mathrm{cos}\left(q\right)-{S}_{a}\left({l}_{h}\mathrm{sin}\left({q}_{a}\right)+{l}_{v}\mathrm{cos}\left({q}_{a}\right)\right)\right)}{{l}_{t}\mathrm{s}\mathrm{i}\mathrm{n}\left({q}_{k}-{q}_{h}\right)}+\frac{sin\left({q}_{k}\right)\left({l}_{c}\mathrm{sin}\left(q\right)+{S}_{a}\left({l}_{h}\mathrm{cos}\left({q}_{a}\right)-{l}_{v}\mathrm{sin}\left({q}_{a}\right)\right)\right)}{{l}_{t}\mathrm{s}\mathrm{i}\mathrm{n}\left({q}_{k}-{q}_{h}\right)}$$

$$\:{S}_{k}\triangleq\:\frac{sin\left({q}_{h}\right)\left({l}_{c}\mathrm{cos}\left(q\right)-{S}_{a}\left({l}_{h}\mathrm{sin}\left({q}_{a}\right)+{l}_{v}\mathrm{cos}\left({q}_{a}\right)\right)\right)}{{l}_{l}\mathrm{s}\mathrm{i}\mathrm{n}\left({q}_{k}-{q}_{h}\right)}+\frac{sin\left({q}_{h}\right)\left({l}_{c}\mathrm{sin}\left(q\right)+{S}_{a}\left({l}_{h}\mathrm{cos}\left({q}_{a}\right)-{l}_{v}\mathrm{sin}\left({q}_{a}\right)\right)\right)}{{l}_{l}\mathrm{s}\mathrm{i}\mathrm{n}\left({q}_{k}-{q}_{h}\right)}$$

respectively. Solving (4) for $\:{S}_{h}$ and $\:{S}_{k}$ requires matrix inversion that can only be done if $\:{q}_{h}+{q}_{k}\ne\:n\pi\:$, $\:n\in\:\mathbb{Z}$, meaning the bicycle should be adjusted such that the hindlimb of the rat never reaches full extension.

The principle of virtual work can be used to develop a relationship between the torque produced at each joint $\:j\in\:\mathcal{J}\triangleq\:\left\{\mathrm{h}\mathrm{i}\mathrm{p},\:\mathrm{k}\mathrm{n}\mathrm{e}\mathrm{e},\:\mathrm{a}\mathrm{n}\mathrm{k}\mathrm{l}\mathrm{e}\right\}$ and the resultant torque at the crank^38^. The work done at the crank $\:{\dot{W}}_{\mathrm{c}\mathrm{r}\mathrm{a}\mathrm{n}\mathrm{k}}:\:Q\to\:\mathbb{R}$ can be expressed as $\:{\dot{W}}_{\mathrm{c}\mathrm{r}\mathrm{a}\mathrm{n}\mathrm{k}}={\tau\:}_{\mathrm{c}\mathrm{r}\mathrm{a}\mathrm{n}\mathrm{k}}\dot{q}$, where $\:{\tau\:}_{\mathrm{c}\mathrm{r}\mathrm{a}\mathrm{n}\mathrm{k}}$ is the torque at the crank. The work done at the crank caused by the work at each joint $\:{\dot{W}}_{\mathrm{c}\mathrm{r}\mathrm{a}\mathrm{n}\mathrm{k}}^{j}$is equal to the work done at each joint. The total work done at the crank is $\:{\dot{W}}_{\mathrm{c}\mathrm{r}\mathrm{a}\mathrm{n}\mathrm{k}}={\sum\:}_{j\in\:\mathcal{J}}\:{\dot{W}}_{\mathrm{c}\mathrm{r}\mathrm{a}\mathrm{n}\mathrm{k}}^{j}$. The work done at the hip can be written as

$$\:{\dot{W}}_{\mathrm{c}\mathrm{r}\mathrm{a}\mathrm{n}\mathrm{k}}^{\mathrm{h}\mathrm{i}\mathrm{p}}={\tau\:}_{\mathrm{h}\mathrm{i}\mathrm{p}}{S}_{h}\dot{q}$$

**Fig. 3:**
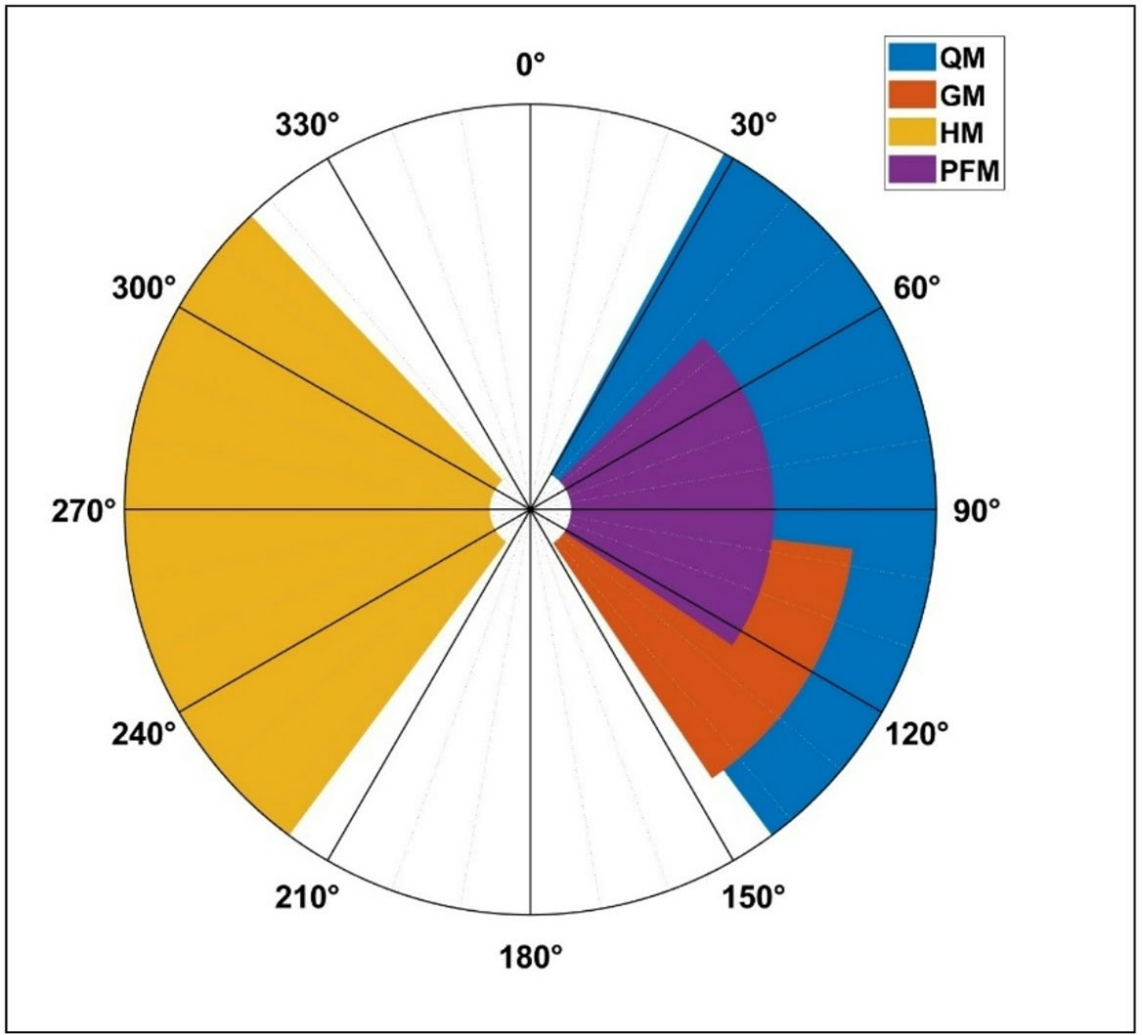
The stimulation pattern found for the right leg muscles as a function of the crank angle *q* to produce clockwise rotation.

where $\:{\tau\:}_{\mathrm{h}\mathrm{i}\mathrm{p}}$is the torque produced by the hip. Similarly, the work at the knee and ankle are $\:{\dot{W}}_{\mathrm{c}\mathrm{r}\mathrm{a}\mathrm{n}\mathrm{k}}^{\mathrm{k}\mathrm{n}\mathrm{e}\mathrm{e}}={\tau\:}_{\mathrm{k}\mathrm{n}\mathrm{e}\mathrm{e}}\left(-{S}_{h}+{S}_{k}\right)\dot{q}$ and $\:{\dot{W}}_{\mathrm{c}\mathrm{r}\mathrm{a}\mathrm{n}\mathrm{k}}^{\mathrm{a}\mathrm{n}\mathrm{k}\mathrm{l}\mathrm{e}}={\tau\:}_{\mathrm{a}\mathrm{n}\mathrm{k}\mathrm{l}\mathrm{e}}\left({S}_{h}-{S}_{k}+{S}_{a}\right)\dot{q}$, respectively. Based on the principle of virtual work, torque transfer ratios $\:{T}_{j}:\:Q\to\:\mathbb{R}$ that relate the torque at each joint $\:j\in\:\mathcal{J}\triangleq\:\left\{\mathrm{h}\mathrm{i}\mathrm{p},\:\mathrm{k}\mathrm{n}\mathrm{e}\mathrm{e},\:\mathrm{a}\mathrm{n}\mathrm{k}\mathrm{l}\mathrm{e}\right\}$ to the resultant torque at the crank can be expressed as^38^

5 $$\:{T}_{\mathrm{h}\mathrm{i}\mathrm{p}}\left(q\right)={S}_{h}$$

6 $$\:{T}_{\mathrm{k}\mathrm{n}\mathrm{e}\mathrm{e}}\left(q\right)={-S}_{h}+{S}_{k}$$

7 $$\:{T}_{\mathrm{a}\mathrm{n}\mathrm{k}\mathrm{l}\mathrm{e}}\left(q\right)={S}_{h}-{S}_{k}+{S}_{a}$$

### Model-guided development of stimulation patterns

Two kinematic models were developed. The first model (Version A) assumed that independent control over four muscle groups – gluteal muscle (GM), quadriceps muscle (QM), hamstrings muscle (HM), and plantarflexor muscle (PFM) groups – was possible. The second model (Version B) assumed that independent control over the muscle groups was not possible but that independent control over the femoral and sciatic nerves was possible.

#### Version A: four muscle stimulation pattern

To delay fatigue onset, which can be detrimental to continuous cycling, FES duration should be minimized, and each muscle should be stimulated only when the contraction of that muscle produces sufficiently large forward motion about the crank. Assuming that biarticular effects of each muscle group can be neglected, the torque transfer ratios in (5)-(7) can be translated to stimulation regions denoted $\:{\mathcal{Q}}_{m}\subset\:\mathcal{Q}$ for each $\:m\subset\:\mathcal{M}\triangleq\:\{\mathrm{G}\mathrm{M},\:\mathrm{Q}\mathrm{M},\:\mathrm{H}\mathrm{M},\:\mathrm{P}\mathrm{F}\mathrm{M}\}$, corresponding to the GM, QM, HM, and PFM groups. When viewed from a position lateral to the limb receiving FES, GM stimulation causes clockwise torque at the hip (hip extension); QM stimulation causes counterclockwise torque at the knee (knee extension); HM stimulation causes clockwise torque at the knee (knee flexion); and PFM stimulation causes clockwise torque at the ankle (plantarflexion). Multiplying the torque transfer ratios in (5)-(7) by the active torques at each joint, respectively, gives the resultant torque at the crank caused by the FES-induced contraction of each muscle. Because forward pedaling requires clockwise motion of the crank, the GM should only be stimulated when $\:{T}_{\mathrm{h}\mathrm{i}\mathrm{p}}^{S}$ is positive. Similarly, the QM should only be stimulated when $\:{T}_{\mathrm{k}\mathrm{n}\mathrm{e}\mathrm{e}}^{S}$ is negative, the HM when $\:{T}_{\mathrm{k}\mathrm{n}\mathrm{e}\mathrm{e}}^{S}$ is positive, and the PFM when $\:{T}_{\mathrm{a}\mathrm{n}\mathrm{k}\mathrm{l}\mathrm{e}}^{S}$ is positive. Additionally, to prevent muscles from being stimulated at points when the torque transfer ratio is near-zero, we introduce a user-selected constant $\:{\epsilon\:}_{m}\in\:{\mathbb{R}}_{>0}$ for each $\:m\in\:\mathcal{M}$ so that each muscle is only stimulated when the magnitude of its torque transfer ratio is sufficiently large to cause forward motion of the crank. These constants should be selected such that $\:{\epsilon\:}_{m}<\mathrm{m}\mathrm{a}\mathrm{x}\left(\left|{T}_{j}\right|\right)$ so that each muscle group receives FES at some point in the crank cycle. The stimulation regions are defined for each muscle group as

8 $$\:{\mathcal{Q}}_{\mathrm{G}\mathrm{M}}\triangleq\:\left\{q|{T}_{\mathrm{h}\mathrm{i}\mathrm{p}}\left(q\left(t\right)\right)>{\epsilon\:}_{\mathrm{G}\mathrm{M}}\right\}$$

9 $$\:{\mathcal{Q}}_{\mathrm{Q}\mathrm{M}}\triangleq\:\left\{q|{-T}_{\mathrm{k}\mathrm{n}\mathrm{e}\mathrm{e}}\left(q\left(t\right)\right)>{\epsilon\:}_{\mathrm{Q}\mathrm{M}}\right\}$$

10 $$\:{\mathcal{Q}}_{\mathrm{H}\mathrm{M}}\triangleq\:\left\{q|{T}_{\mathrm{k}\mathrm{n}\mathrm{e}\mathrm{e}}\left(q\left(t\right)\right)>{\epsilon\:}_{\mathrm{H}\mathrm{M}}\right\}$$

11 $$\:{\mathcal{Q}}_{\mathrm{P}\mathrm{F}\mathrm{M}}\triangleq\:\left\{q|{T}_{\mathrm{a}\mathrm{n}\mathrm{k}\mathrm{l}\mathrm{e}}\left(q\left(t\right)\right)>{\epsilon\:}_{\mathrm{P}\mathrm{F}\mathrm{M}}\right\}$$

Stimulation to each muscle group $\:m\in\:\mathcal{M}$ is activated if $\:q\in\:{\mathcal{Q}}_{m}$. Otherwise, FES to that muscle group is turned off. The developed hindlimb kinematic equations were simulated over one crank cycle using MATLAB (Ver. R2023a; The MathWorks, Inc). An example of the developed switching pattern for the muscle stimulation of the right leg can be seen in Fig. 3, where the different colored wedges correspond to the stimulation of the indicated muscle group in that region of the crank cycle.

#### Version B: two nerve stimulation pattern

Due to the small sizes of the individual hindlimb muscles of the rat and their close proximity to each other, it can be difficult to independently stimulate individual muscle groups using non-invasive or minimally-invasive techniques. Moreover, near-complete overlap existed between the modelled QM region of activity (RoA) and the RoA for the GM in kinematic model Version A, suggesting that independent GM stimulation may not be required. Under this scenario, independent stimulation of the femoral and sciatic nerves would elicit contractions in the anterior (QM) and posterior (HM, GM, and PFM) hindlimb groups, respectively. Thus, kinematic model Version B was developed to account for this scenario.

As above, the torque transfer ratios in (5)-(7) can be translated to stimulation regions denoted $\:{\mathcal{Q}}_{n}\subset\:\mathcal{Q}$ for each $\:n\subset\:\mathcal{N}\triangleq\:\{\mathrm{f}\mathrm{n},\:\mathrm{s}\mathrm{n}\}$, where fn and sn correspond to the muscles innervated by the femoral and sciatic nerves, respectively. Stimulation of the femoral nerve elicits contractions in the QM group, so the stimulation region for the femoral nerve, denoted $\:{\mathcal{Q}}_{\mathrm{f}\mathrm{n}}$ should be applied in $\:{\mathcal{Q}}_{\mathrm{q}\mathrm{u}\mathrm{a}\mathrm{d}}$ in (9). Stimulation of the sciatic nerve causes co-contractions of the GM, HM, and PFM groups, so further analysis is required to establish the stimulation $\:{\mathcal{Q}}_{\mathrm{s}\mathrm{n}}$.

**Fig. 4:**
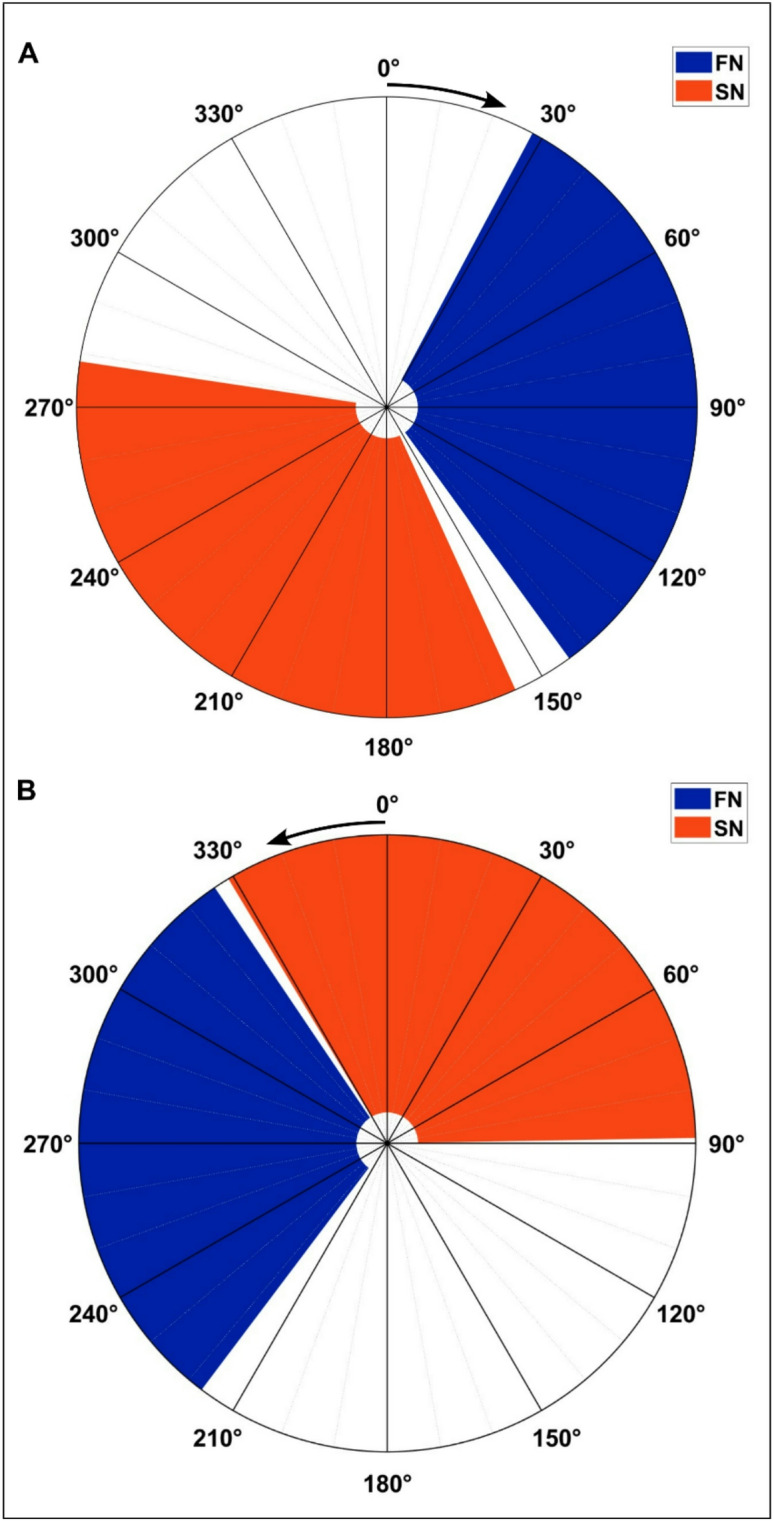
The stimulation pattern found for the right leg nerves as a function of the crank angle *q* to produce clockwise (A) and counterclockwise (B) rotation.

Stimulation of the GM can only cause clockwise torque at the hip, the QM cause counterclockwise torque at the knee, the HM cause clockwise torque at the knee, and the PFM cause clockwise torque at the ankle. By multiplying the torque transfer ratios in (5)-(7) by the active torques at each joint, respectively, gives the resultant torque at the crank caused by the stimulation of each muscle. Because forward pedaling requires, clockwise motion of the crank, the GM group should only be stimulated when $\:{T}_{\mathrm{h}\mathrm{i}\mathrm{p}}^{S}$ is positive. Similarly, the QM group should only be stimulated when $\:{T}_{\mathrm{k}\mathrm{n}\mathrm{e}\mathrm{e}}^{S}$ is negative, the HM when $\:{T}_{\mathrm{k}\mathrm{n}\mathrm{e}\mathrm{e}}^{S}$ is positive, and the PFM when $\:{T}_{\mathrm{a}\mathrm{n}\mathrm{k}\mathrm{l}\mathrm{e}}^{S}$ is positive. Additionally, to prevent muscles from being stimulated at points when the torque transfer ratio is near-zero, we introduce a user-selected constant $\:{\epsilon\:}_{m}\in\:{\mathbb{R}}_{>0}$ for each $\:m\in\:\mathcal{M}$ so that each muscle is only stimulated when the magnitude of its torque transfer ratio is sufficiently large to cause forward motion of the crank. These constants should be selected such that $\:{\epsilon\:}_{m}<\mathrm{m}\mathrm{a}\mathrm{x}\left(\left|{T}_{j}\right|\right)$ so that each muscle group receives FES at some point in the crank cycle. The stimulation regions are defined for each muscle group as

12 $$\:{\mathcal{Q}}_{\mathrm{f}\mathrm{n}}\triangleq\:\left\{q|-{T}_{\mathrm{k}\mathrm{n}\mathrm{e}\mathrm{e}}\left(q\left(t\right)\right)>{\epsilon\:}_{\mathrm{f}\mathrm{n}}\right\}$$

13 $$\:{\mathcal{Q}}_{\mathrm{s}\mathrm{n}}\triangleq\:\left\{q|{T}_{\mathrm{c}\mathrm{r}\mathrm{a}\mathrm{n}\mathrm{k}}>{\epsilon\:}_{\mathrm{s}\mathrm{n}}\right\}$$

where $\:{T}_{\mathrm{c}\mathrm{r}\mathrm{a}\mathrm{n}\mathrm{k}}={a}_{1}{T}_{\mathrm{h}\mathrm{i}\mathrm{p}}+{a}_{2}{T}_{\mathrm{k}\mathrm{n}\mathrm{e}\mathrm{e}}+{a}_{3}{T}_{\mathrm{a}\mathrm{n}\mathrm{k}\mathrm{l}\mathrm{e}}$ and $\:{a}_{1},\:{a}_{2},\:{a}_{3}\in\:{\mathbb{R}}_{>0}$ are known constants. Stimulation to each nerve $\:n\in\:\mathcal{N}$ is activated if $\:q\in\:{Q}_{n}$. Otherwise, FES to that nerve is turned off. The developed hindlimb kinematic equations were simulated over one crank cycle using MATLAB. An example of the developed switching pattern for the nerve stimulation of the right leg can be seen in Fig. 4, where the different colored wedges correspond to the stimulation of the indicated nerve in that region of the crank cycle.

### Model validation

#### Ethical approval & husbandry

To validate the kinematic model, nine experiments were performed over 110 days with Sprague-Dawley rats (*N* = 2, one male, one female) starting at 4 months of age under a protocol approved by the Institutional Animal Care and Use Committee (IACUC) at Malcom Randall Veterans Affairs Medical Center (MRVAMC). All experiments were performed in accordance with the relevant guidelines and regulations set forth by Public Health Service, the Animal Welfare Act, the Guide for Care and Use of Laboratory Animals, and ARRIVE. Animals were purchased from Charles River Labs and housed at the MRVAMC. While not undergoing experiments, rats were maintained on a standard 12:12 light: dark cycle. Water and standard rat chow were provided *ad libitum*.

#### Stimulus titration

At the start of an experiment, the rat was anesthetized with isoflurane (1.5% with O2 flow at 1000 mL/min) and was placed on a 37 °C heated water blanket to assist in temperature regulation. This approach was used to ensure that the kinematic model was validated in a neurally intact rat and that the animal experienced no pain or distress during the initial testing and validation of our kinematic model. Ophthalmic ointment was applied to prevent drying. The right hindlimb was shaved. The rat was positioned over the bicycle and placed in a sling that was attached to a horizontal rod above the bicycle (“R” in Fig. 1), like in previous papers^27–29^. Care was taken to ensure the sling did not interfere with the motion of the lower limbs. The hip was positioned to be vertically in-line with the crank arm axis. After alignment, the tail was fixed to the horizontal rod to further reduce body movement during pedaling. Once the rat was positioned correctly, the right hind paw was securely fixed to the right pedal with adhesive strips to prevent the foot from slipping off the pedal. In comparison, the left limb was gently supported from the rod using adhesive strips so that the left foot did not touch the left pedal. An infrared clip was attached to the left foot and used to monitor heart rate, breathing rate, and blood oxygenation via a MouseOx Plus pulse oximeter (Starr Life Sciences). Body temperature was continuously monitored.

Prior to titrating the stimulation, the QM and HM of the right hindlimb were located by palpation during limb manipulation. Using a pair of sterile monopolar needle electrodes (Technomed, 0.45 mm x 26 g) connected to a current-controlled stimulator (NeuroControl), locations were determined that targeted the femoral and sciatic nerves, resulting in robust contraction of the target muscle groups. After confirming the target with the needle electrodes, the needle electrodes were removed and a pair of fine wire electrodes (Rhythmlink, 50 mm x 25 g) were introduced at the same locations.

Using micrograbbers attached to the fine wire electrodes, electrical stimulation was delivered to the rat using a computer-controlled stimulator (Hasomed P24 Science). The pedals were positioned prior to each test and placed in a location where electrical stimulation was expected to lead to an advantageous rotation (robust rotation in the clockwise direction) based on the kinematic model. For femoral nerve testing, the pedal was positioned at approximately 90° and for sciatic nerve testing, the pedal was positioned at approximately 180º. Using a systematic staircase method, operational pulse width, pulse amplitude and stimulus frequencies were determined.

#### Mapping activity regions

The femoral nerve and sciatic nerve were stimulated separately using the titrated parameters. The pedal was positioned at a desired starting angle. During a 3s FES timeframe, the change in angle of the crank resulting from muscle contraction was recorded using the optical encoder. The test was repeated at least two more times. The procedure was then repeated at other angles between 0° and 360° in 15° increments in a randomized order. In addition to angular deviation, active plantarflexion or dorsiflexion was noted. Following each stimulus trial, no stimulation was applied for at least 60s to reduce the potential effect of muscle fatigue. After completing tests for one nerve, tests for the other nerve commenced. During each session, nerves were tested in a randomized order. The resulting data were used to calculate a RoA. To find a RoA, the region that captured 95% of observations in which FES resulted in motion, defined as a crank deviation of at least 10°, was determined using observations in which the stimulus frequency was 100 Hz due to the more robust contraction, greater development of torque, and more predictable motion that FES developed. Using the RoA of the experimental data as truth and the RoA of the model as prediction, true positive (TP), true negative (TN), false positive (FP), and false negatives (FN) were calculated as

14 $$\:\mathrm{T}\mathrm{P}=\left({\mathrm{R}\mathrm{o}\mathrm{A}}_{\mathrm{M}}\cap\:{\mathrm{R}\mathrm{o}\mathrm{A}}_{\mathrm{E}}\right)$$

15 $$\:\mathrm{T}\mathrm{N}={\left({\mathrm{R}\mathrm{o}\mathrm{A}}_{\mathrm{M}}\cup\:{\mathrm{R}\mathrm{o}\mathrm{A}}_{\mathrm{E}}\right)}^{{\prime\:}}$$

16 $$\:\mathrm{F}\mathrm{P}=\left({\mathrm{R}\mathrm{o}\mathrm{A}}_{\mathrm{M}}\cap\:{\mathrm{R}\mathrm{o}\mathrm{A}}_{\mathrm{E}}^{{\prime\:}}\right)$$

17 $$\:\mathrm{F}\mathrm{N}=\left({\mathrm{R}\mathrm{o}\mathrm{A}}_{\mathrm{M}}^{{\prime\:}}\cap\:{\mathrm{R}\mathrm{o}\mathrm{A}}_{\mathrm{E}}\right)$$

Using these definitions, the Sensitivity, Specificity, Positive Predictive Value (PPV), and Negative Predictive Value (NPV) were calculated as

18 $$\:\mathrm{S}\mathrm{e}\mathrm{n}\mathrm{s}\mathrm{i}\mathrm{t}\mathrm{i}\mathrm{v}\mathrm{i}\mathrm{t}\mathrm{y}=\frac{\mathrm{T}\mathrm{P}}{\mathrm{T}\mathrm{P}+\mathrm{F}\mathrm{N}}$$

19 $$\:\mathrm{S}\mathrm{p}\mathrm{e}\mathrm{c}\mathrm{i}\mathrm{f}\mathrm{i}\mathrm{c}\mathrm{i}\mathrm{t}\mathrm{y}=\frac{\mathrm{T}\mathrm{N}}{\mathrm{T}\mathrm{N}+\mathrm{F}\mathrm{P}}$$

20 $$\:\mathrm{P}\mathrm{P}\mathrm{V}=\frac{\mathrm{T}\mathrm{P}}{\mathrm{T}\mathrm{P}+\mathrm{F}\mathrm{P}}$$

21 $$\:\mathrm{N}\mathrm{P}\mathrm{V}=\frac{\mathrm{T}\mathrm{N}}{\mathrm{T}\mathrm{N}+\mathrm{F}\mathrm{N}}$$

#### Sensitivity to shifts in body position

To evaluate sensitivity to slight shifts in body position, three scenarios were tested that encompassed the full range in which an adult rat is expected to be operational on the bicycle system: (1) the hip was centered over the crankshaft ($\:{l}_{x}=0$ in Fig. 2); (2) the hips were positioned 1 cm behind the crankshaft ($\:{l}_{x}=-1\mathrm{c}\mathrm{m}$ in Fig. 2); (3) the hips were positioned 1 cm in front of the crankshaft ($\:{l}_{x}=1\mathrm{c}\mathrm{m}$ in Fig. 2). The protocol established for *Mapping RoAs* was followed for each condition. The results for each condition were compared to the kinematic model adjusted for that condition.

## Results

### Stimulus titration

In early pilot experiments, we found that we could easily elicit a robust contraction using a pulse width fixed at 125 µs and stimulus frequency at 100 Hz while varying the pulse amplitude. This was used as a starting point during titration. During brief (< 10s) trials, we determined the pulse amplitude needed to elicit a robust muscle contraction and forward-clockwise (as viewed from the right side of the bicycle) rotation of the crank arm. After the first several experiments produced similar outcomes, a consistent pulse amplitude of 4 mA was used.

### Mapping regions of activity (RoAs)

**Fig. 5:**
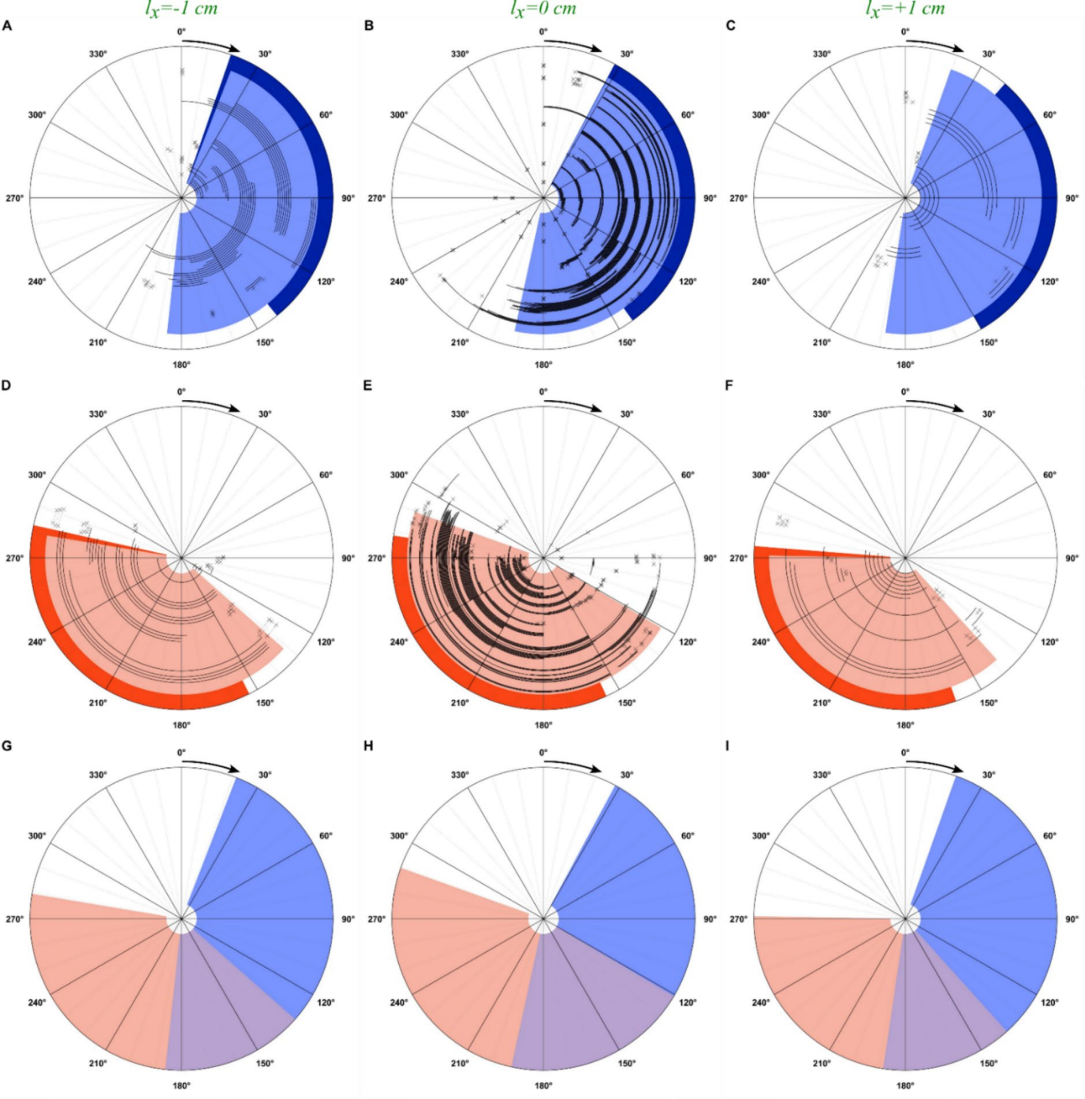
Observed rotation patterns relative to the model predictions. Row 1 (**A, B, C**): femoral nerve. Row 2 (**D, E, F**): sciatic nerve. The hips were shifted 1 cm behind (Left Column;** A, D, G**), 1 cm in front of (Right Column;** C, F, I**), or aligned with the crankshaft (Middle Column;** B, E, H**). Black tracts denoted FES-induced clockwise-forward motion. Locations that resulted in no motion are noted with an “x.” The predictions from the model are shaded darker at the periphery. The lighter, inner shaded region is the estimated region of activation (RoA) based on animal data. Row 3 (**G, H, I**): The estimated RoA for the femoral (blue) and sciatic (orange) nerves showing overlapping regions (purple). Arrow at 0° shows direction of rotation.

**Fig. 6:**
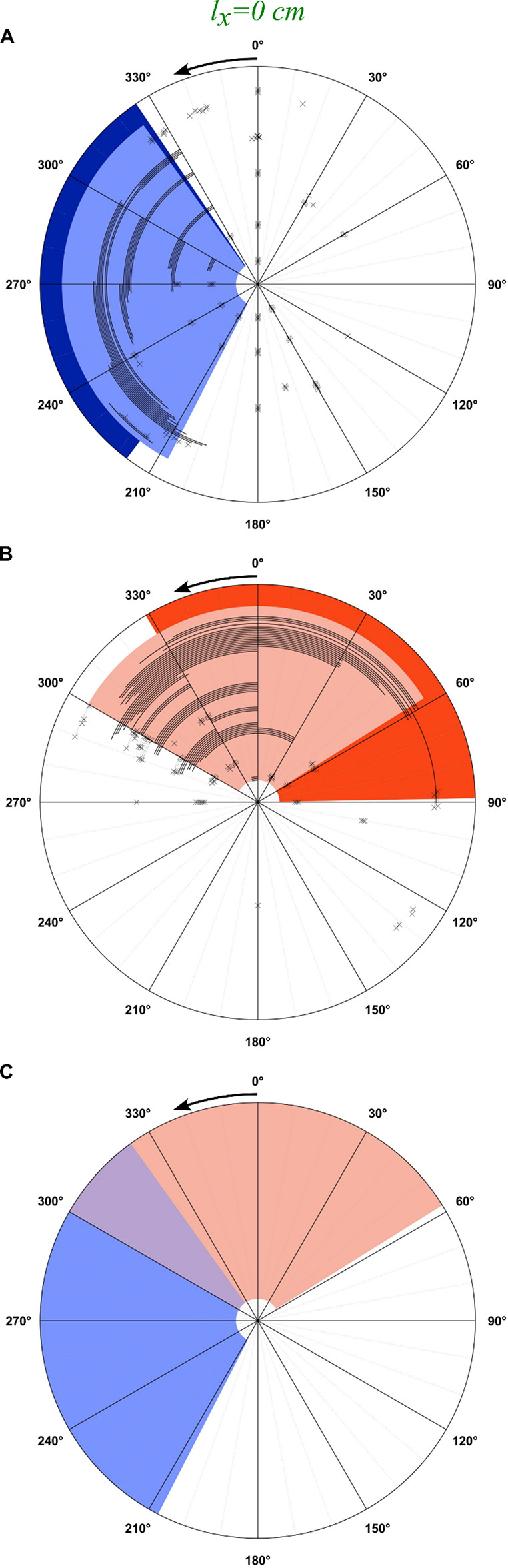
Observed rotation patterns relative to the model predictions for the femoral nerve (**A**) and the sciatic nerve (**B**). The hips were aligned with the crankshaft. Black tracts denoted FES-induced counterclockwise-backwards motion. Locations that resulted in no motion are noted with an “x.” The predictions from the model are shaded darker at the periphery. The lighter, inner shaded region is the estimated region of activation (RoA) based on animal data. (**C**): The estimated RoA for the femoral (blue) and sciatic (orange) nerves showing overlapping regions (purple). Arrow at 0° shows direction of rotation.

A total of 743 trials were conducted in two animals over 9 experiments that occurred over 110 days. Results from mapping the response to femoral and sciatic nerve stimulation are shown in Figs. 5A-F and 6A-B. Each trial is shown as a black arc. The approximated RoA is shaded in light blue (femoral nerve) or light orange (sciatic nerve) while the corresponding model-derived activation region is shown in dark blue and dark orange at the periphery of each panel, respectively. The RoA was defined as the region that captured 95% of observations in which FES resulted in motion of the crankshaft of at least 10°. RoA was developed using observations in which the stimulus frequency was 100 Hz due to the more robust contraction, greater development of torque, and more predictable motion that FES developed (*N* = 449). The majority of data (*N* = 566) were gathered with the hips of the rat centered over the crankshaft (Fig. 5, center column; Fig. 6), with a smaller number of observations at forward (*N* = 57) and backward (*N* = 120) shifts. The effects of a rearward 1 cm shift relative to the crankshaft (Fig. 5, left column) and a forward 1 cm shift relative to the crankshaft (Fig. 5, right column) are also presented, with figures labeled as described above.

In general, the RoA from the experimental data agreed with the RoA from the model (Table 1). The sensitivity – a measure of how well the model identifies true positives – averaged 77.0% ± 8.9% (64.5–91.7%) across both nerves, the three lateral positions, and the two rotation directions. The specificity – a measure of how well the model identifies true negatives – averaged 97.7% ± 4.3% (87.2–100%). The PPV – a measure of the probability that a positive prediction from the model is correct – averaged 95.6% ± 8.7% (74.2–100%). The NPV – a measure of the probability that a negative prediction from the model is correct – averaged 85.4% ± 6.9% (80.2–96.1%).

**Table 1:** Agreement between model and experimental data for clockwise and Counterclockwise motion.

Motion	Clockwise						Counterclockwise	
Shift	Backward		Centered		Forward		Centered	
l_x_ (cm)	-1.0		0.0		+ 1.0		0.0	
Nerve	Femoral	Sciatic	Femoral	Sciatic	Femoral	Sciatic	Femoral	Sciatic
Sensitivity	72.6%	85.1%	70.6%	73.1%	64.5%	83.1%	91.7%	75.4%
Specificity	98.9%	98.3%	99.9%	100.0%	100.0%	98.2%	99.3%	87.2%
PPV	98.3%	97.3%	98.8%	100.0%	100.0%	96.5%	98.4%	74.2%
NPV	81.0%	90.5%	80.2%	80.8%	76.1%	90.8%	96.1%	87.9%

## Discussion

Persons with neurologic injury exhibit reductions in muscle size and function due, in part, to the CNS insult and the resulting disuse^39^. Moreover, neurologic injury predisposes to bone loss and increased fracture risk in the impaired limbs, which is particularly evident in the paralyzed extremities after severe SCI^2,3^ and in hemiparesis following stroke^40^. The etiology of these musculoskeletal declines has resulted in an emphasis on physical rehabilitation modalities (e.g., FES cycling) that use electrical stimulation to activate peripheral nerves and produce muscle contractions against a progressive external resistance in a manner that augments the damaged CNS^15^. FES modalities have been shown to improve muscle recovery in some persons with neurologic injury, when performed with sufficient frequency and duration^5^. However, the ability of FES to restore BMD in the paralyzed limbs is contentious. For example, a few clinical trials have reported that FES cycling produced relatively minor trabecular BMD improvement when performed 4–5 days/week, 30–60 min per bout for 6–12 months, while others report no trabecular or cortical bone benefits^1^. As such, the need exists to optimize FES parameters to increase BMD and bone strength and to ensure that such regimens also continue to improve muscle recovery. Approaching this issue purely from a clinical trials perspective would necessitate multiple years-long studies to design optimal regimens for bone recovery, given the many factors that can be varied when designing FES schemes (e.g., frequency and duration of training sessions, pedaling cadence, and the frequency, amplitude, and pulse width of electrical stimulation, among others), and the slow rate of bone accrual that occurs in people^14^. Herein, we detail kinematic models and stimulation patterns that are needed to facilitate the development of closed-loop FES hindlimb cycling in rats, a model species that exhibits similar mechanisms of bone loss and regain as humans^16^. Within the context of severe contusion SCI, the rat model has been shown to reproduce the complex pathophysiology of severe traumatic SCI in humans^17^, along with the deleterious bone phenotype that occurs in humans with severe traumatic SCI^18–24^. However, the rate of bone loss and regain occurs much faster in rodent SCI models^18,19^ than in humans after SCI^2,25^, potentially allowing for more high-throughput testing and identification of effective FES schemes. Previous kinematic and stimulation pattern analyses have been performed for lower-extremity muscle groups in human FES-cycling^34^, but the quadrupedal locomotion of rats results in unique body position, hindlimb kinematics, and joint angles^35^ that require different considerations than humans. The theoretical kinematic models we developed were assessed and validated through experimental trials on anesthetized young to middle-aged adult male and female rats, akin to the largest proportion of persons experiencing SCI in both the US military^41^ and general populations^42^, providing an initial foundation to develop FES patterns for use in closed-loop controllers that facilitate continuous FES-cycling in adult rats across the age-span. Such systems can be used to better understand how FES schemes impact bone, muscle, and other health parameters in preclinical models, including those of neurologic injury.

The overall purpose of this study was to determine the feasibility of using a kinematic modelling analysis, like that used in human FES-cycling^34^, to characterize appropriate crankshaft RoAs where FES would contribute to forward (clockwise) pedaling and positions where FES may be counterproductive, such as producing no pedaling motion or counterclockwise pedaling. When FES produced clockwise rotation of at least 10°, it was considered productive motion. All other outcomes - when FES produced rotation greater than 0° but less than 10°, when it produced counterclockwise motion, or when it produced no motion (“dead zones”) – were considered counterproductive. In doing so, our original muscle-based kinematic analysis included four hindlimb muscle groups: QM (knee extensors), HM (knee flexors), GM (hip extensors), and PFM. The estimated QM RoA to produce forward pedaling using kinematic analysis Version A ranged from 30° to ~ 140°, with estimated GM and PFM RoAs ranging from ~ 45° to 140°. These findings indicated near-complete overlap in estimated RoAs for GM and PFM when compared with QM, suggesting that simultaneous GM and PFM stimulation would contribute minimally beyond that of QM stimulation. In comparison, the estimated HM RoA to produce forward pedaling ranged from ~ 220° to 315°, with no overlap amongst other muscle groups evaluated.

To assess the validity of these models, we initially attempted to use surface electrodes to deliver transcutaneous electrical stimulation to each individual muscle group (data not shown), as is typical within human FES-cycling. However, we were unable to obtain sufficient precision with this approach. The relatively small size of the rat hindlimb muscles in relation to the surface electrodes as well as their proximity of each muscle resulted in stimulus spillover and simultaneous recruitment of muscle groups that precluded pedaling motion. Additionally, because rat skin is relatively loose and mobile, we observed considerable movement of the electrode in relation to the underlying nerves and muscles during pedaling. As such, data obtained from surface electrodes were not included in this study and instead the included data were limited to those obtained with fine wire percutaneous electrodes, which represents a more precise approach to stimulate nerves and muscle groups of interest. Importantly, the precision to place the fine wires into small muscles to achieve independent and repeatable control is difficult, so we elected to use fine wires to recruit the femoral and sciatic nerves instead.

We then devised a modified nerve-based kinematic analysis (Version B) that included only femoral nerve or sciatic nerve stimulation, which could be assessed and validated in real-world experiments using indwelling fine wire electrodes placed in precise muscle locations. Our nerve-based kinematic analysis indicated femoral nerve FES would produce forward pedaling from 30° to ~ 140°, like our original model, and that sciatic nerve FES would produce forward pedaling from ~ 160° to ~ 280°. To assess the validity of these models, we completed several FES trials in which we selectively stimulated the femoral nerve or sciatic nerve in anesthetized rats (1 male and 1 female) on different days over a series of months and across different crankshaft angles to identify appropriate and counterproductive RoAs. In doing so, we observed consistent forward pedaling throughout the estimated RoAs predicted in our nerve-based model, with 98–100% specificity. However, several differences also existed between the estimated RoAs and our real-world data. For example, in sciatic nerve FES, our real-world data indicated that forward pedaling initiated at ~ 125°, which was prior to the estimated RoA in our model. Likewise, in femoral nerve FES, our real-world data indicated that forward pedaling continued beyond 140° to ~ 190°, which was after the estimated RoA end-range in our model. These differences in sensitivity are likely explained by a constraint used in our nerve-based kinematic model that did not permit RoA overlap between the femoral and sciatic nerves. This model constraint eliminated the possibility of simultaneous femoral and sciatic nerve stimulation that would result in co-contraction of antagonist knee extensor/flexor and hip extensor/flexor muscle groups, exacerbate muscle fatigue, and ultimately prove deleterious to continuous forward pedaling. As such, our real-world experiments appear to validate the estimated femoral and sciatic nerve RoAs that were predicted in our nerve-based kinematic analysis.

While conducting our real-world trials, we noted several observations that should be considered when developing closed-loop control systems to automate rat FES cycling. For example, vertical and horizontal hip placement varied in relation to the crankshaft between animals, largely due to the differing body sizes and limbs lengths of adult male and female rats. Moreover, within the same rat, we noted slight variations in hip placement across bouts, despite our best efforts to maintain identical positioning, which never exceeded ± 1 cm horizontal translation from the crankshaft due to rat limb length limitations. We consider these variations inherent to the design of the bicycle system. To systematically assess how hip position might impact femoral and sciatic nerve RoAs, we altered the inputs in our nerve-based kinematical analysis to place the hips either 1 cm forward or rearward of the crank, which represented the maximum real-world difference (based on limb lengths) that permitted rats to perform cycling without limb overextension. In doing so, we observed only slight changes in the estimated RoAs for each nerve, which did not appreciably impact the modeled RoAs. We subsequently verified these estimated RoAs in real-world trials in which the hips were carefully positioned forwards or rearwards of the cranks and noted no perceivable impact on sensitivity of specificity.

In addition, we noted minor differences in FES start and stop angles across trials in the same rats, even when carefully controlling hip position. In comparison, within each testing session considerable consistency in FES start and stop angles was noted. One potential explanation for the differences across trials was slightly different fine wire electrode placement across sessions, which may impact the degree to which muscles are recruited in response to femoral or sciatic nerve stimulation. In this regard, femoral nerve FES recruits all QM, with each individual muscle (vastus lateralis, vastus medialis, vastus intermedius, and rectus femoris) producing knee extension, while the rectus femoris also produces hip flexion, which must be overcome to allow forward pedaling. To address this, future studies could tattoo the skin to identify exact reproducible locations for fine wire electrode placement across trials or could consider surgical implantation of nerve cuffs on the femoral and sciatic nerves to allow identical nerve stimulation across trials. The former may still result in variations among trials as rat skin can move significantly relative to the target muscle, particularly when significant atrophy occurs like after SCI^43^. In comparison, the latter would result in greater consistency, but would involve an invasive surgical procedure that may independently impact muscle and/or bone recovery and increase the risk of infection. Regardless, the 700 plus FES trials performed on the rat bicycle for the femoral and sciatic nerves verified that our kinematic models accurately predicted RoAs for each nerve that, when stimulated, produced consistent and meaningful forward pedaling motions with 98–100% specificity.

Though not tested explicitly, these data suggest that it should be possible to traverse the entire rotational field with bilateral independent stimulation of the femoral and sciatic nerves. In this bilateral 4-nerve scenario, nearly 75% of the unit circle can be traversed by stimulating one of two nerves, as denoted by overlapping RoAs. This redundancy should delay the onset of fatigue and/or allow for greater torque development.

As with all studies, several potential limitations also warrant mention. First, our kinematic models were developed using average limb length inputs for 4-months-old (adult) male rats and were not re-developed in an age- or sex-specific manner. As such, actual RoAs may differ for smaller or larger rats. Regardless, we tested an adult male and female rat with differing body sizes and limb lengths over a series of months and observed similar femoral and sciatic nerve RoAs and forward pedaling responses across animals, when using an identical FES pulse width (125 µs), stimulation frequency (100 Hz), and variable pulse amplitude. This suggests that the limb length inputs in our kinematic analyses were robust to differences in body size and limb length, at least in adult rats over 4-months of age. Moreover, limb length values are input variables in our kinematic model that can be selectively varied in future experiments, if considerably smaller or larger animals are tested. Second, when testing FES, we fixed pulse width and stimulation frequency and varied pulse amplitude, as noted above, based on a preliminary titration experiment that indicated these parameters produced robust muscle contractions. However, these FES parameters were not optimized to produce forward pedaling, to limit fatigue, or to induce any physiologic adaptations. As such, future studies are needed to systematically optimize FES parameters to achieve the desired outcome within a closed-loop control system and to discern whether bone or muscle adaptations occur. Third, we incorporated 60-second rest periods between successive nerve stimulation bouts to lessen potential fatigue that could prove deleterious to forward pedaling, but we did not measure neuromuscular fatigue to verify that this rest period was sufficient to achieve this outcome. Regardless, the considerable consistency observed in FES start and end angles across successive femoral and sciatic nerve bouts suggested the existence of minimal fatigue. Lastly, we verified femoral and sciatic nerve RoAs using fully anesthetized rats to validate our kinematic model in neurally intact animals and to ensure animals experienced no pain or distress during testing. As such, it remains to be determined whether the FES parameters used to validate these RoAs are appropriate for unanesthetized rats or whether these stimulation parameters will differ in models of SCI or other neurologic injuries that experience considerable muscle atrophy and neuromuscular impairment. Regardless, the experiments and RoAs described herein provide an initial foundation to develop closed-loop control systems that are capable of measuring crank angle and that automatically adjust stimulation parameters to ensure consistent forward pedaling, which are key factors needed to optimize FES-cycling schemes for physiologic benefit.

## Conclusion

A passive bicycle system for rats was developed and corresponding FES patterns for the femoral and sciatic nerves were simulated, tested, and validated on live animals. These FES patterns will enable future work aimed at the design of closed-loop controllers for a rat FES-cycling system. Ultimately, these studies may provide cost- and time-effective means to identify favorable preclinical FES parameters for bone and muscle restoration and may assist in improving other health outcomes that are negatively impacted in response to neurologic injury.

## Data Availability

The datasets generated and analyzed during the current study will be made available in the Harvard Dataverse repository upon acceptance of the manuscript and are available upon request. Additionally, HMS or WED can be contacted for data pertaining to the kinematic models while MAS and JFY can be contacted for data from *in vivo* experiments.
